# The Expression of AQP1 IS Modified in Lung of Patients With Idiopathic Pulmonary Fibrosis: Addressing a Possible New Target

**DOI:** 10.3389/fmolb.2018.00043

**Published:** 2018-05-03

**Authors:** Ana Galán-Cobo, Elena Arellano-Orden, Rocío Sánchez Silva, José Luis López-Campos, César Gutiérrez Rivera, Lourdes Gómez Izquierdo, Nela Suárez-Luna, María Molina-Molina, José A. Rodríguez Portal, Miriam Echevarría

**Affiliations:** ^1^Departamento de Fisiología Médica y Biofísica, Instituto de Biomedicina de Sevilla, Hospital Universitario Virgen del Rocío, CSIC, Universidad de Sevilla, Sevilla, Spain; ^2^Unidad Médico-Quirúrgica de Enfermedades Respiratorias, Hospital Universitario Virgen del Rocio, Sevilla, Spain; ^3^Centro de Investigación Biomédica en Red sobre Enfermedades Respiratorias (CIBERES), Madrid, Spain; ^4^Servicio Anatomía Patológica, HU Virgen del Rocío Sevilla, Seville, Spain; ^5^Laboratorio de Neumologia Experimental, Servicio de Neumologia, Institut d'Investigació Biomédica de Bellvitge, Hospital Universitario de Bellvitge, Barcelona, Spain

**Keywords:** interstitial lung disease (ILD), fibrosis, sarcoidosis, aquaporins (AQPs), inflamation, type II pneumocytes, IPF, AQP1

## Abstract

Activation of the epithelial-mesenchymal transition process (EMT) by which alveolar cells in human lung tissue undergo differentiation giving rise to a mesenchymal phenotype (fibroblast/miofibroblasts) has been well recognized as a key element in the origin of idiopathic pulmonary fibrosis (IPF). Here we analyzed expression of AQP1 in lung biopsies of patients diagnosed with IPF, and compared it to biopsies derived from patients with diverse lung pneumonies, such as hypersensitivity pneumonitis, sarcoidosis or normal lungs. Immunostaining for AQP1 showed a clear increment of AQP1 localized in the alveolar epithelium in biopsies from IPF patients alone. Moreover, to examine the possible participation of AQP1 in the pathophysiology of IPF, we evaluated its role in the pro-fibrotic transformation induced by transforming growth factor (TGF-β) *in vitro*. Human alveolar epithelial cells (A549), and fibroblasts derived from an IPF patient (LL29), or fibroblasts from healthy normal lung tissue (MRC-5), were treated with TGF-β, and levels of expression of AQP1, as well as those of E-cadherin, vimentin, α-SMA and collagen were analyzed by RT-qPCR, western blot and immunohistochemistry. An increase of AQP1 mRNA and protein after TGF-β treatment (4–72h) was observed either in A549 or IPF fibroblast-LL29 but not in MRC-5 fibroblasts. A gradual reduction of E-cadherin, and increased expression of vimentin, with no changes in α-SMA levels were observed in A549. Whereas in LL29 and MRC-5, TGF-β1 elicited a large production of collagen and α-SMA that was significantly greater in IPF fibroblast-LL29. Changes observed are consistent with activation of EMT by TGF-β, but whether modifications in AQP1 expression are responsible or independent events occurring at the same time is still unknown. Our results suggest that AQP1 plays a role in the pro-fibrotic TGF-β action and contributes to the etiology and pathophysiology of IPF. Understanding AQP1's role will help us comprehend the fate of this disease.

## Introduction

Idiopathic pulmonary fibrosis (IPF) is the most widely found interstitial lung disease. IPF is a progressive disease usually fatal, characterized by inability of the alveolar epithelium to repair a lesion. The re-epithelialization fails and transformation to fibroblast/myofibroblast persist, with increase in extracellular matrix deposition and alteration of functional lung architecture, which will finally produce a respiratory failure (Lynch et al., 2006; Raghu et al., 2011). Clinical IPF is normally associated in surgical lung biopsy with a histopathological pattern of usual interstitial pneumonia (UIP). Though the origin of this pathology remains not fully understood, (Willis and Borok, 2007; Spagnolo et al., 2015) it is postulated that following alveolar epithelial injury, a process of repair and scar formation is initiated that implicates important changes and cell activation. However, in some cases, an alteration of the healing process occurs and lung fibrosis develops (Willis et al., 2006). In IPF, activated epithelial cells and recruited inflammatory cells release potent fibrogenic growth factors and cytokines that favor fibroblasts proliferation and its transformation to myofibroblasts (Khalil et al., 2005; Takai et al., 2013). The activation of an epithelial-mesenchymal transition (EMT) process by which alveolar epithelial cells (AEC) undergo a transition or trans-differentiation process to mesenchymal like cells, such as fibroblasts or myofibroblasts, is gaining acceptance as a key element in the origin of this pathology. Both pathological alternative pathways do not exclude each other and may likely coexist in the origin of IPF.

Different fibrogenic growth factors have been clearly implicated as inducers of lung fibrosis, among them the transforming growth factor (TGF-β) is perhaps the most relevant factor associated to lung fibrosis. Furthermore, a crucial role for TGF-β has been indicated in the induction of EMT in many physiological scenarios, including for instances in tissue fibrosis (Chapman, 2011; Tirino et al., 2013). It has been demonstrated that TGF-β induces *in vitro* EMT in AEC, and that epithelial and mesenchymal markers co-localize in hyperplastic alveolar type II cells in interstitial pulmonary fibrosis (IPF) tissue, indicating that AEC could show great plasticity and function as a source for new fibro- and myofibroblasts cells in lung fibrosis (Chapman, 2011; Tirino et al., 2013).

Several aquaporins (AQP1, AQP3, AQP4, and AQP5) have been recognized in the respiratory system. Each one presents in a particular location, probably indicating different physiological roles in the pulmonary tissue (Guarino et al., 2009; Camelo et al., 2014). AQP1 is expressed in both membranes, apical and basolateral, of the microvascular endothelium, besides of being expressed in the visceral pleura. AQP3 and AQP4 are expressed in the airway epithelium. AQP5 is expressed in the apical membrane of type-I pneumocytes, and also in the apical side of acinar cells in the submucosal glands of nasopharynx and rest of airways.

In previous studies, we demonstrated that Aquaporin-1 (AQP1) up-regulates its expression in the lung of animals exposed to hypoxia for 24–48 h (Echevarría et al., 2007). We also demonstrated that the transcription factor inducible by hypoxia HIF-1a, partially participates in this process along with other factors in the inflammatory pathway (Abreu-Rodríguez et al., 2011). We then became interested if the regulation by hypoxia would occur in patients suffering hypoxemic diseases and whether the remodeling of AQP1 expression may play a role in developing pathologies, such as chronic obstructive pulmonary disease (EPOC) (Calero et al., 2014) and IPF (Gutiérrez et al., 2013).

A clear association between expression of AQP1 and increment of cell proliferation in different cellular models has been recently demonstrated (Galán-Cobo et al., 2015, 2016). Moreover, in a murine model of acute lung injury a relationship between ANGII and AQP1 expression was indicated (Cao et al., 2010) and up-regulation of pulmonary AQP1 expression was observed in mice with pulmonary fibrosis induced by bleomycin (Gao et al., 2013), leading authors to propose a possible role for AQP1 in the pathogenesis of lung fibrosis.

Thus, with all these precedents in mind we decided to look for alternative pathways that may explain connections between changes in the AQP1 expression pattern in lung cellular types of patients with IPF and the pathophysiology of the disease. Lung biopsies of patients with different interstitial lung disorders were analyzed by immunohistochemistry for AQP1 expression and experiments in cell lines treated with the pro-fibrotic agent TGF-β1 were performed *in vitro*. The human alveolar epithelial cell line A549, and two different cell lines of human fibroblast, one derived from an IPF patient (LL-29) and the other from healthy fibroblast (MRC5), were treated with TGF-β1, and the correlation between expression of AQP1 and other proteins were analyzed to determine their role in the induced EMT or fibroblast-myofibroblast transition process.

## Materials and methods

### Human tissue samples

All paraffin-embedded samples were acquired from the Department of Pathology, HUVR of Seville, Spain. Originally, samples were obtained by pulmonary biopsy corresponding to IPF and other fibrotic and non-fibrotic interstitial lung diseases (ILD). For the control group we took samples of patients undergoing surgery for spontaneous pneumothorax otherwise healthy. The number of groups, as well as the number of samples included per group in the analysis are indicated in Table 1, and are as follow: (20) idiopatic pulmonary fibrosis-usual interstitial pneumonia (IPF-UIP), (2) non-specific interstitial pneumonia (NSIP), (1) cryptogenic organizing pneumonia (COP), (5) IPF-not otherwise specified, (6) hypersensitivity pneumonitis, (4) sarcoidosis and (7) control lungs. Written informed consent was obtained from all participants. The study followed the tenets of the WMA Declaration of Helsinki for research involving human subjects and was approved by the ethics committee of the University Hospital Virgen del Rocío (HUVR).

**Table 1:** Summary of interstitial lung diseases (pneumonias) included in the study.

	**N**	**(%)**
Idiopathic Pulmonary Fibrosis (IPF)/Usual interstitial pneumonia (IPF-UIP)	20	44.4
Non-specific interstitial pneumonia (NSIP)	2	4.4
Cryptogenic organizing pneumonia (COP)	1	2.2
Idiopathic interstitial pneumonia not otherwise specified	5	11.1
Hypersensitivity pneumonia	6	13.3
Sarcoidosis	4	8.9
Controls	7	15.6
Total	45	100

### Immunohistochemistry of biopsies

All samples examined were obtained from formalin-fixed, paraffin-embedded pieces. Slices of 5 μm were cut with a microtome and mounted on microscope slides. Immunohistochemical samples were obtained from paraffin sections that were immersed in xylene and rehydrated through a series of decreasing dilutions of ethanol. Blocking of endogenous peroxidase, epitope retrieval and immunostaining procedure were done as previously reported (López-Campos et al., 2011). Primary antibody, rabbit polyclonal anti-AQP1, (1:500 dilution, Abcam, Cambridge, UK) was used overnight, for the developing of brown precipitates we then used the two steps system EnVision + Dual Link System-HRP (DakoCytomation, Dako Denmark). Qualitative analysis was made by two independent observers. We assigned zero for no staining on the sample, low positive (<25% of the sample stained), medium positive (50% of the sample stained) and high positive (>75% of the sample stained) values (Tables 1, 2). Immunoreactivity was analyzed in terms of surface and type of cells showing AQP1 on their surface. Samples were photographed using an AX70-Olympus microscope equipped with an Olympus DP10 camera (Denmark).

**Table 2:** Association between AQP1 staining and interstitial pneumonias.

	**Type II pneumocytes AQP1 immunoreactivity**				*P*-value^*^
	**Negative**	**Low positive**	**Medium positive**	**High positive**	
Usual interstitial pneumonia (*n* = 20)	2 (10%)	1 (5%)	8 (40%)	9 (45%)	0.001
Non-specific interstitial pneumonia (*n* = 2)	–	–	1 (50%)	1 (50%)	0.087
Cryptogenic organizing pneumonia (*n* = 1)	–	–	1 (100%)	–	0.249
Idiopathic interstitial pneumonia not otherwise specified (*n* = 5)	4 (80%)	–	1 (20%)	–	0.685
Hypersensivity pneumonia (*n* = 6)	3 (50%)	3 (50%)	–	–	0.266
Sarcoidosis (*n* = 4)	4 (100%)	–	–	–	1
Control (*n* = 7)	6 (85.7%)	1 (14.3%)	–	–	–

*Two independent observers gave negative value for no stain, low positive for < 25% of the sample stained, medium positive for 50% of the sample stained and high positive for >75% of the sample stained*.

* *P-value was calculated by χ2 with Monte Carlo method for association between AQP1 staining and interstitial pneumonias comparing each interstitial pneumonia against the control group and using the Bonferroni correction for multiple comparisons (p < 0.008) only the group of usual interstitial pneumonia resulted different from control (p = 0.001)*.

### Cell culture

A549 cells (Human lung alveolar epithelial) and LL29 (Human idiopathic pulmonary fibrosis) were purchased from the American Type Culture Collection (ATCC, Rockville, USA); MRC-5 (Human lung fibroblast cell line) cells were obtained from the European Collection of Cell Culture (ECACC, Salisbury, UK). A549 cells were cultured as monolayers in DMEM:F12 (F12K Nut Mix) supplemented with 10% heat-inactivated fetal bovine serum, 100 U/mL penicillin-streptomycin (Invitrogen, Carlsbad, CA, USA). MRC-5 cells were cultured as monolayers in MEM supplemented with 10% heat-inactivated fetal bovine serum, 100 U/mL penicillin-streptomycin, and 2 mM L-glutamine (Invitrogen, Carlsbad, CA, USA). LL-29 cells were cultured in Ham's F12K medium supplemented with 15% heat-inactive fetal bovine serum, 100 U/mL penicillin-streptomycin, and 2 mM L-glutamine (Invitrogen, Carlsbad, CA, USA). Cells were cultured at 5% CO_2_ at 37°C.

### TGF-β1 assays

A549 (2.4 × 10^5^), MRC-5 (3 × 10^5^), and LL-29 (2.5 × 10^5^) cells were plated on 60 mm dishes (Nunc, Roskilde, Denmark), when the cells reached 80% of confluence we added fresh medium 1% FBS for 24 h and then we added 10 ng/ml of TGF-β1 (R&D System, Minneapolis, MN, USA) for 4, 16, 24, 48, or 72 h. We used untreated cells as control.

Morphological changes in A549, MRC-5 and LL-29 cells were examined by inverted fluorescence microscopy (Zeiss Axiovert 25, Carl Zeiss Co. Oberkochen, Germany) and photographed using a CCD Canon camera.

### Indirect immunocytochemistry staining of cultures cell

Cells were seeded into 24-well plates at a concentration of 2 × 10^4^ cells/well. After treatment with 10 ng/ml TGF-β1 for 24 h the culture medium was discarded and cells were washed with Ca^++^-Mg^++^ PBS 1x and fixed with 3% paraformaldehyde for 10 min at room temperature. Cells were washed and permeabilized/blocked with 0.1% Triton X-100 in 10% Fetal Calf Serum (FCS) solution for 10 min at room temperature and incubated with primary antibodies; 1:200 of E-cadherin (BD Biosciences, San Jose, CA, USA) and 1:100 of AQP1 (Abcam, Cambridge, UK) overnight at 4°C. After washing with PBS cells were then incubated with Alexa 488 donkey anti-mouse (Invitrogen, Carlsbad, CA, USA) 1:200 in blocking solution for AQP1 and E-cadherin staining for 1 h at room temperature. F-actin was labeled with red-fluorescent Alexa Fluor 594 Phalloidin (Thermo Fisher Scientific, Waltham, MA, USA) 1:1000 for 10 min at room temperature. Samples were washed with PBS and then mounted with DAKO (Invitrogen, Carlsbad, CA, USA) and preserved at 4°C. Immunocytochemestries were photographed using a fluorescence microscope Olympus BX 71 with a refrigerated camera DP70.

### Western blotting

Plated cells were washed with cold PBS, collected by scraping in 1 ml of cold PBS and centrifuged at 300 g for 5 min at 4°C. For whole-cell protein extract, the pellet was lysed in a variable volume of homogenization buffer containing: 50 mM Hepes (pH 7.3); 5 mM EDTA; 250 mM NaCl; 5 mM DTT; 0.2% (v/v) NP40 (Sigma–Aldrich); and 1% (v/v) Complete Protease Inhibitors Cocktail (Sigma–Aldrich). The resuspended pellet was left on ice for 5–15 min, vortexed, and then centrifuged at 16,000 g for 15 min at 4°C, and proteins contained in the supernatant were stored. Protein concentration was analyzed using the Bradford method (BioRad Protein Assay, BioRad, Berkeley, CA) and kept at −20°C until Western blot analysis (Galán-Cobo et al., 2016). For all proteins analyzed, 30 μg of whole-cell extracts were resolved by SDS-PAGE on 10–12% gels. After electrophoresis, proteins were transferred onto PVDF membranes (Hybond-P, Amersham Biosciences, Pittsburgh, PA) using a Novex apparatus (Novel Experimental Technology, San Diego, CA). Membranes were probed with 1:700 anti-AQP1 (Abcam, Cambridge, UK), 1:2500 anti-E-Cadherin (BD Biosciences, San Jose, CA, USA), 1:1500 anti-Vimentin (Abcam, Cambridge, UK), 1:1000 anti-α-SMA (Abcam, Cambridge, UK) and 1:10000 anti-cyclophilin A (Abcam, Cambridge, UK) antibodies. Immunoreactive bands were developed with the ECL Prime system (Amersham Biosciences) and visualized using a digital imaging system (ImageQuant LAS 4000 Mini, GE Healthcare, Buckinghamshire, UK).

### RNA isolation and quantitative PCR

RNA was extracted from cells using TRIzol reagent (Invitrogen, Carlsbad, CA, USA) according with manufacturer protocol. After mRNA isolation, reverse transcription (RT) was performed for 5 μg of RNA using SuperScript II RNase-H reverse transcriptase (Invitrogen, Carlsbad, CA, USA). Real-time quantitative PCR (qPCR) analysis was carried out in an ABI Prism 7500 Sequence Detection System using SYBR Green PCR Master Mix (Applied Biosystems, Warrington, UK) and the thermocycler conditions recommended by the manufacturer. The expression of HPRT1 was used to normalize for differences in amounts of input DNA between samples. Primers were designed using the Primer Express software (version 2.0, Applied Biosystems, Warrington, UK) and their sequences are indicated in Table 3. Melting curve analysis showed a single sharp peak with the expected melting temperature for all samples.

**Table 3:** Primer sequences used for the amplification of cDNAs by qPCR.

**Genes**	**Forward sequence**	**Reverse sequence**
Aquoporin 1 (*AQP1*)	GGACACCTCCTGGCTATTGACTAC	GTTGCTGAAGTTGTGTGTGATCAC
E-Cadherin (*CDH1*)	TCGACACCCGATTCAAAGTG	GTCCCAGGCGTAGACCAAGA
Vimentin (*VIM*)	TGCCCTTAAAGGAACCAATGAG	AGGCGGCCAATAGTGTCTTG
α-SMA (*ACTA2*)	CTGTTCCAGCCATCCTTCAT	CCGTGATCTCCTTCTGCATT
Collagen Type I Alpha 1 (*COL1A1*)	TGACCGAGACGTGTGGAAAC	CAGATCACGTCATCGCACAAC
Hypoxanthine Phosphoribosyltransferase 1 (*HPRT1*)	ACTGAACGTCTTGCTCGAGATG	AGCAGGTCAGCAAAGAATTTATAGC

*Primers were designed using the Primer Express software (Applied-Biosystems). qPCR: quantitative PCR*.

### Statistical analysis

Data analysis was performed using SPSS Advanced Statistics (SPSS Inc., Chicago, Illinois), version 19.0. For the statistical analysis of data derived from analysis by immunohistochemistry of patient biopsies, the “*p*” value for comparative analysis of association between AQP1 staining and interstitial pneumonias was calculated by χ^2^ with Monte Carlo method. The Bonferroni correction was used for multiple comparisons and the Fisher exact test when comparing each interstitial pneumonia against the control group. For all experiments performed in cells in culture the data are expressed as mean ± standard error of the mean from at least four different experiments. The statistical significance for normal distribution data was estimated using one-way ANOVA followed by Bonferroni's *post-hoc* test to compare more than two groups. Data with non-normal distribution were analyzed using Mann-Whitney U or Kruskal-Wallis H-test for two or more than two groups, respectively. Values of *p* ≤ 0.05 were considered significant.

## Results

### AQP1 is specifically expressed in hyperplasic cuboidal type II pneumocytes in the alveolar epithelium in patients with idiopathic pulmonary fibrosis

**Figure 1:**
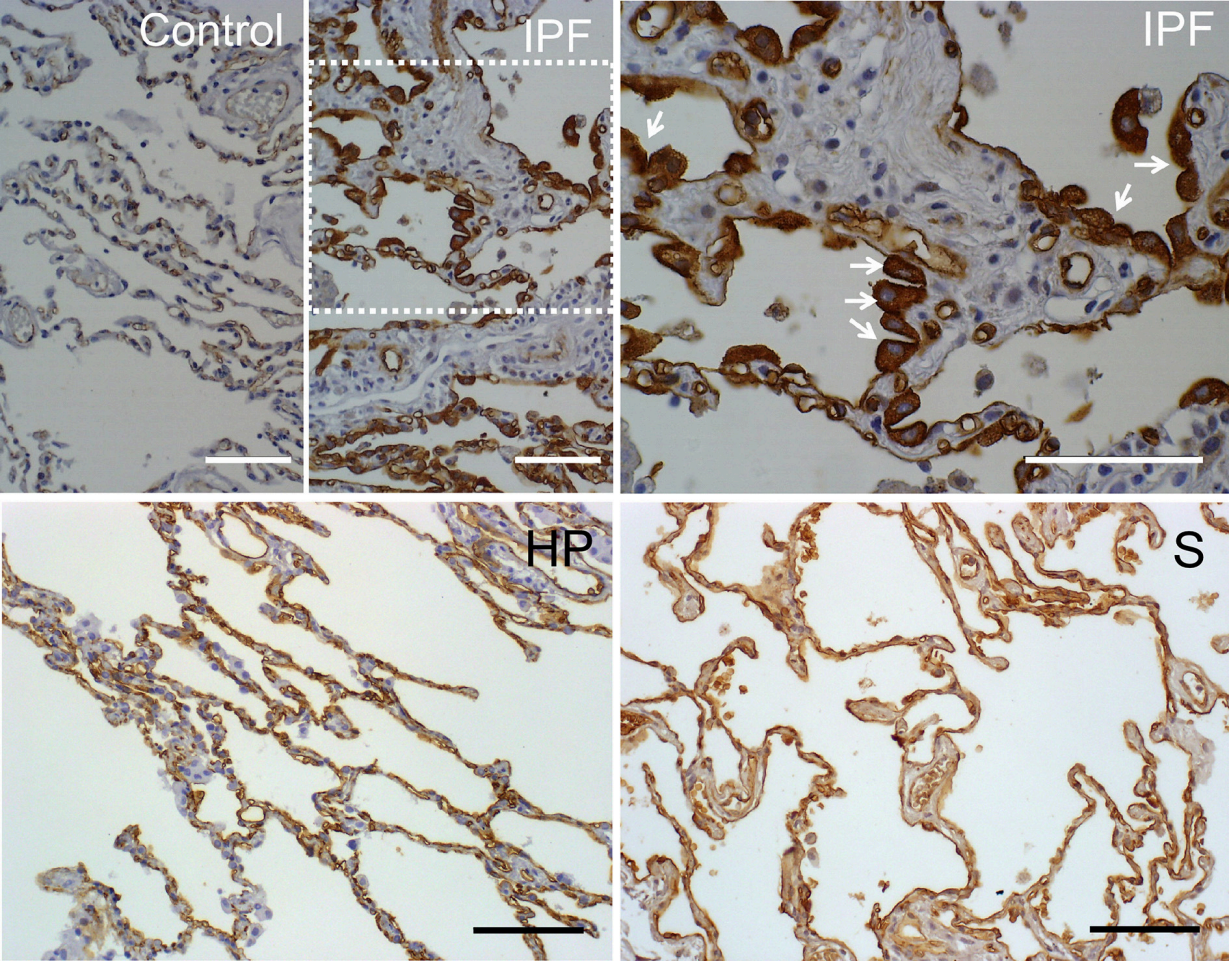
Immunostaining of AQP1 in lung biopsies of patients. Pulmonary parenchyma of a healthy patient is shown as control. In the pulmonary parenchyma of patients with usual intersticial pneumonia (IPF) reactive type II pneumocytes of alveolus showed intense expression of AQP1 (see the arrows in the IPF at larger magnification). In biopsies of patients with hypersensitivity pneumonia (HP) or sarcoidosis (S), the staining of AQP1 was mainly located in capillaries endothelia. Scale bar represents 100 μm in each case. Note the different size of bar in each case.

In the present study we analyzed expression of AQP1 in lung biopsies of patients diagnosed with IPF, and compare it to biopsies derived from diverse interstitial lung diseases such as others pneumonias, hypersensitivity pneumonitis, sarcoidosis, or normal lungs (Table 1). Conditions for AQP1 antibody immunohistochemistry in human lung biopsies were determined in a previous study (López-Campos et al., 2011). A summary of results from the analysis of biopsies is presented in Table 2. Normal lung tissues is consistent with previous reports (López-Campos et al., 2011; Calero et al., 2014) in that AQP1 immunostaining was detected predominantly in endothelial cells of alveoli capillaries and the rest of blood vessels; faint labeling was also detected on neumocytes of the alveolar wall (Figure 1). No labeling was detected in absence of the primary antibody (data not shown).

Compared to the rest of pathologies analyzed, the immunohistochemistry assays revealed that only biopsies from IPF show a clear increment of AQP1 staining that localizes remarkably over hyperplasic cuboidal type II pneumocytes in the alveolar epithelium, and to a much lesser grade over few planar, hyperplasic or metaplasic type I cells (Figure 1). A more detailed observation over the fibrosis focus of IPF patients, where the transition from fibroblasts to miofibroblasts occurred, showed that type I epithelial cells lining the fibrotic focus are totally deprived of AQP1 expression. In the rest of the samples basal AQP1 expression was limited to erythrocytes and endothelial cells. Absence of AQP1 labeling was observed over the interstitial lung or in myoepithelial cells. Changes in the expression pattern of β-catenin and E-cadherin were also studied but need further analysis of the immunoassays. In conclusion, the analysis demonstrates a possible role for AQP1 expression in the reactive process occurring over fibrotic focus of the alveoli epithelium of IPF patients.

### AQP1 is overexpressed along epithelial mesenchymal transition (EMT) induced by TGF-β

**Figure 2:**
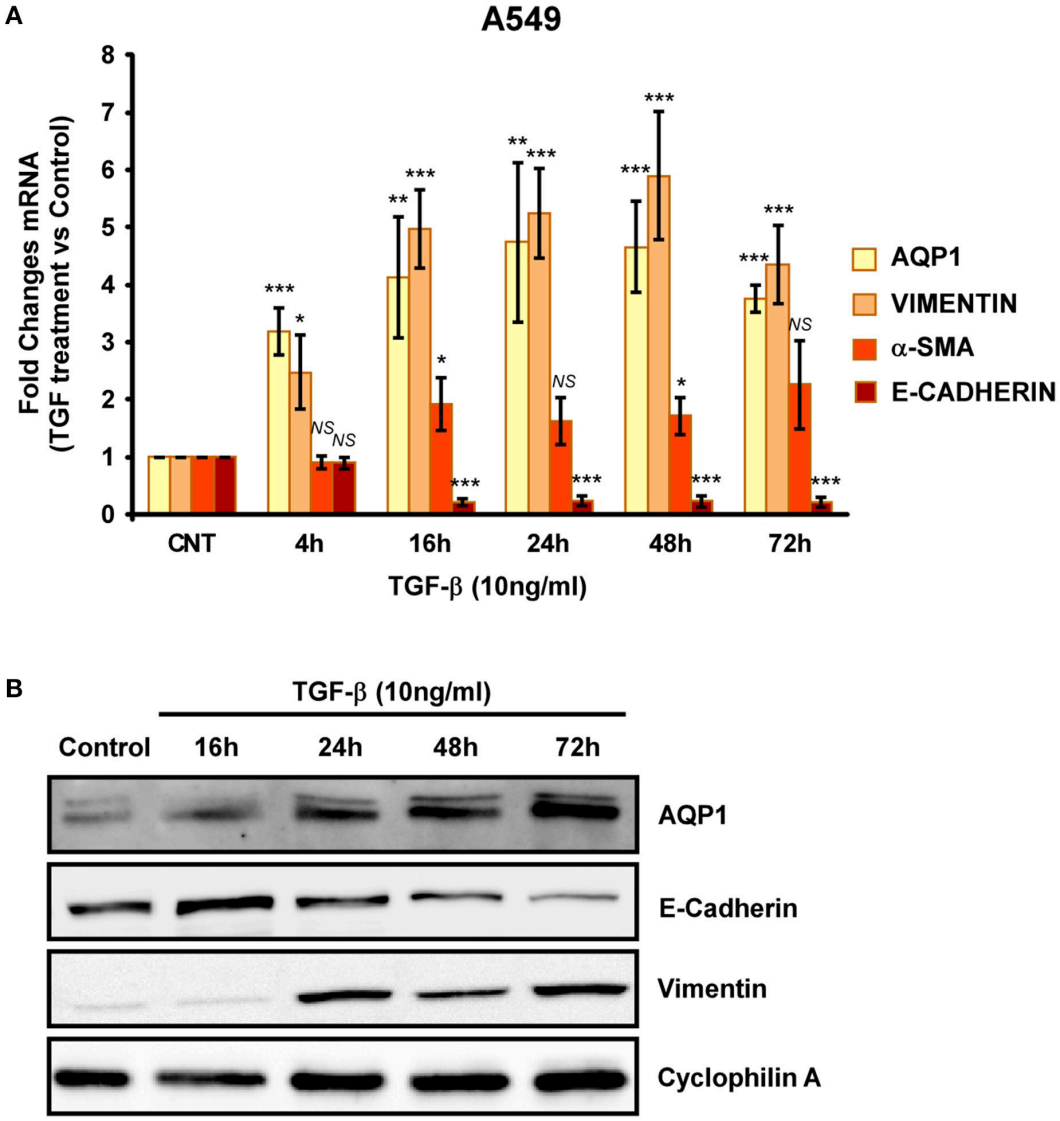
Analysis of EMT induction in A549 cell lines. Cells were treated with TGF-β1 (10 ng/ml) for 4, 16, 24, 48, and 72 hours and the mRNA levels of AQP1, E-cadherin, Vimentin and α-SMA where quantified by real time RT-PCR analysis **(A)** and the protein levels by Western blot **(B)**.

**Figure 3:**
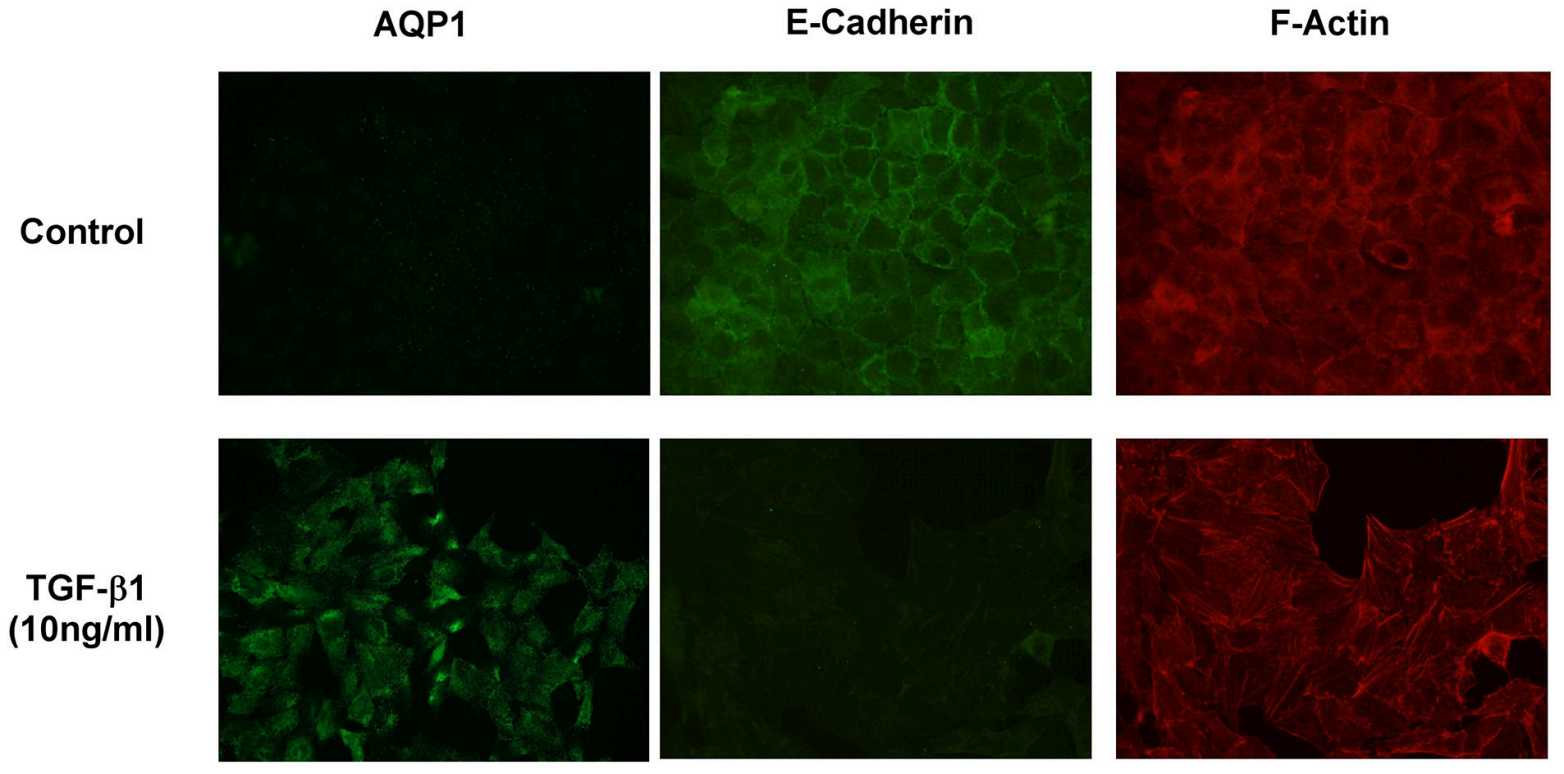
Immunostaining showing AQP1, E-cadherin and F-actin expression in A549 cells without TGF-β1 treatment (Control) and after TGF-β1 (10 ng/ml) treatment.

To further investigate the implication of AQP1 in the initiation and progression of IPF we examined epithelial cells as central to the pathogenesis of IPF. Experiments performed in the human lung epithelial cell line A549 has shown that treatment with the pro-fibrotic agent TGF-β1 (10 ng/ml) produces a time course (24, 48, 72, and 96 h) differentiation of the epithelial cells toward a fibroblastic-like phenotype, together with an increase of mRNA levels of the mesenchymal markers vimentin (5-fold) and α-SMA (2.5-fold). Consequently, a decrease in the levels of the epithelial marker E-cadherin was found after TGF-β1 treatment. Interestingly, levels of AQP1-mRNA were also measured and a substantial increase of AQP1 expression (5-fold) was observed in parallel to the changes described before for vimentin and α-SMA (Figure 2A). Similar results to those described for the mRNA, were found for the protein levels of E-cadherin, vimentin and AQP1 (Figure 2B). Immunostaining for AQP1 again showed higher expression levels of AQP1 in the lung epithelial cells A549 after TGF-β1 treatment (Figure 3), supporting previous data and indicating a possible role of AQP1 in the EMT process in lung tissue.

**Figure 4:**
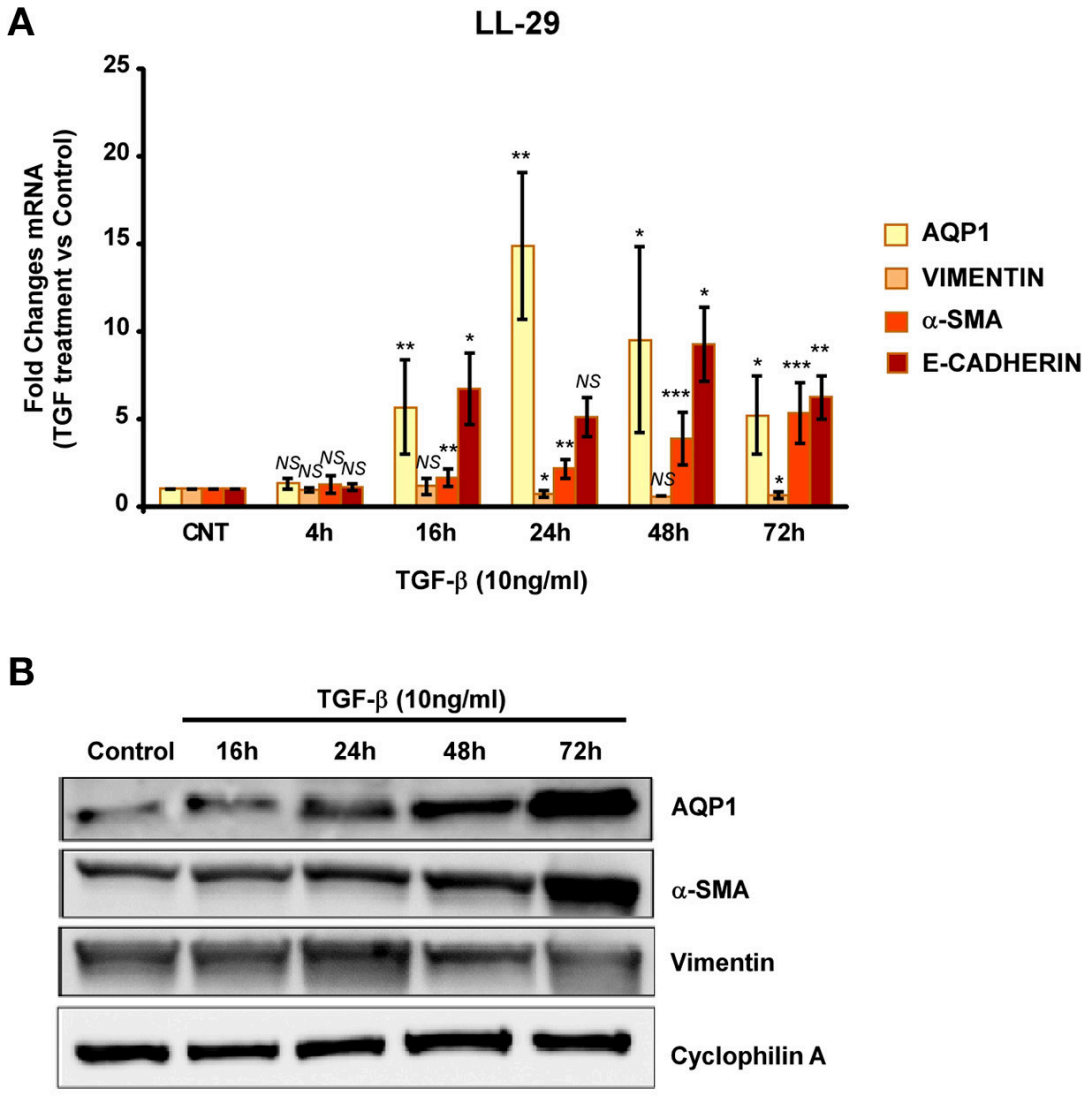
Analysis of fibroblast-myofibroblast transition induction in LL-29 cell line. Cells were treated with TGF-β1 (10 ng/ml) for 4, 16, 24, 48, and 72 hours and the mRNA levels of AQP1, Vimentin, α-SMA and Collagen type I where quantified by real time RT-PCR analysis **(A)** and the protein levels of AQP1, Vimentin and α-SMA were quantified by Western blot **(B)**.

**Figure 5:**
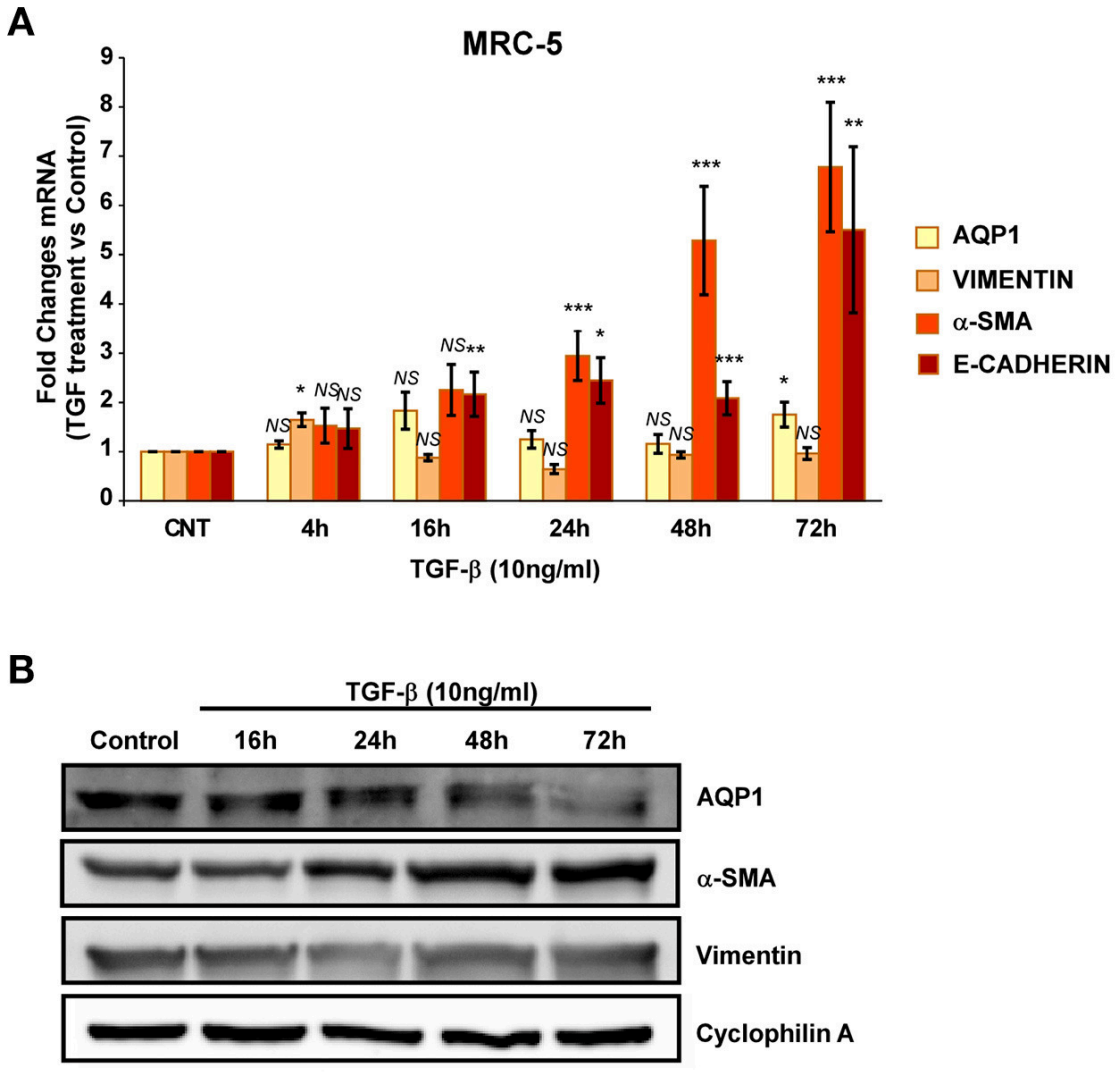
Analysis of fibroblast-myofibroblast transition induction in MRC-5 cell line. Cells were treated with TGF-β1 (10 ng/ml) for 4, 16, 24, 48, and 72 hours and the mRNA levels of AQP1, Vimentin, α-SMA and Collagen type I where quantified by real time RT-PCR analysis **(A)** and the protein levels of AQP1, Vimentin and α-SMA were quantified by Western blot **(B)**.

We then repeated similar experiments with TGF-β1 using two different fibroblast cell lines. A cell line of fibroblasts derived from patients with IPF (LL29) and a cell line of healthy fibroblasts (MRC-5). An increment of the capacity for extracellular matrix production was found in LL29 cells after TGF-β1 treatment indicated by higher levels of type I collagen (10-fold) and of α-SMA (5-fold). In parallel a strong and stable induction of AQP1 mRNA and protein levels were observed in this cell line obtained from an IPF patient (Figure 4) unlike, in the healthy fibroblast cell line MRC-5. Although similar induction of α-SMA and type I collagen were found, AQP1 levels remained unchanged after TGF-β1 treatment (Figure 5).

Altogether, these results support the hypothesis that increments of AQP1 expression induced by TGF-β1 occur in the lung tissue of patients with IPF. As a result of rising AQP1 in the alveolar epithelia, the cells undergo EMT or fibroblast-myofibroblast transition, thereby contributing to formation of fibroblastic foci and subsequent fibroblast and extracellular matrix accumulation.

## Discussion

Immunohistochemical studies in rats and mice have shown that AQP1 is present in the capillaries and in the sub-epithelial connective tissue of the animal's respiratory tract. Alveolar cells do not express AQP1 on its surface and expression of AQP1 is restricted to the capillary endothelium (Nielsen et al., 1997). At present, the idea remains that AQP1 provides the principal pathway for osmotic transport of water between airspace and lung capillary compartments. Nevertheless, neither alveolar fluid clearance in neonatal and adult lung nor lung fluid accumulation in experimental models of lung injury have reported being affected by AQP1 deletion.

In the present work, we have shown for the first time a large expression of AQP1 in human lung tissue from IPF patients, not only in endothelial cells but also in the alveolar epithelial surface. Alveolar surface is covered by two types of epithelial cells, alveolar cells called type I (AT I) and type II (AT II), also called pneumocytes type I and type II. AT I cells represent 90% of the alveolar border and are responsible for making gas exchange. AT II cells are characterized by synthesizing and secreting pulmonary surfactant which actives alveolar surface and prevent its collapse among other important functions such as immune defense (Chroneos et al., 2010). AT II cells are progenitors of AT I cells and therefore are responsible for repairing the alveolar epithelium after damage. Under normal conditions, the alveolar epithelial repair occurs through the proliferation of AT II cells and their differentiation into AT I cells. However, IPF is characterized by the fact that both alveolar type II cells and the type I undergo apoptosis and are replaced by fibroblasts (Barbas-Filho et al., 2001) contributing to the disappearance and transformation of the alveolar epithelium. As previously described, ATI cells do not express AQP1 (Nielsen et al., 1997). Interestingly, our data shows that expression of AQP1 in the alveolar epithelium was exclusively found in ATII cells.

Analysis performed in different diseases other than IPF show that AQP1 expression is not uniform. This water channel is not expressed in normal lung alveolar cells and is expressed to a much lesser extent in AT II cells from samples obtained from various interstitial lung diseases other than IPF. Even in cases of intense inflammatory activity like hypersensitivity pneumonitis or sarcoidosis, there is not expression of AQP1 in alveolar epithelial cells. The highest expression of AQP1 in type II alveolar epithelial cells occurs significantly in IPF samples. IPF is a progressive disease of unknown etiology (Crystal et al., 2002) that classically presents a histopathological pattern of UIP. The primary characteristics are areas of conventional fibrosis with variable regions of fibroblastic foci, scattered with zones of normal or almost normal lung. Additionally, IPF is also typified by ATII cell hyperplasia (Selman and Pardo, 2003). Although initial studies gave special attention and prevalent role to inflammation on fibrogenesis, the present hypothesis proposes a principal role of the epithelium in the pathogenesis and progression of this disease. Thus, IPF is likely the result of an initial epithelial injury to which the natural repair process fail to repair setting an initial focus of cell activation and damage (Chilosi et al., 2003). Identification of cells in which markers of epithelial and mesenchymal phenotypes are co-expressed, seen both in human lung and in cultures *in vitro*, sustain the paradigm by which alveolar epithelial cells may serve as a supply of myofibroblasts in lung fibrosis (Willis et al., 2005). Currently, activation and apoptosis of epithelial cells is considered one of the initial events in the development of IPF (Sisson et al., 2010).

In IPF, proliferation and migration are two important events in the alveolar epithelium transformation. Interestingly, AQP1 has been implicated in cell proliferation and migration promotion in lung among other proliferative tissue models (Saadoun et al., 2005; Wei and Dong, 2015; Wu et al., 2015; Galán-Cobo et al., 2016; Tomita et al., 2017; Wang et al., 2017; Yun et al., 2017). On the other hand, EMT is a key event in the IPF progression, which promotes accumulation of fibroblasts and deposition of extracellular matrix in response to epithelial injury. TGF-β signaling is considered a key activator of EMT and fibroblast activation in this disease (Chapman, 2011; Tirino et al., 2013). Migration is an essential process during EMT, and although AQP1 has not been directly related with EMT, it is well know that this protein helps the cell migration process by facilitating water influx at the tip of the lamellipodium, resulting in a membrane protusion (Saadoun et al., 2005), and by interacting with β-catenin (Monzani et al., 2009; Yun et al., 2017). Other AQPs, such as AQP3 and AQP5, activate this process as well (Chen et al., 2014, 2017). Therefore, the large increase in expression of AQP1 we observed on AT II cells in IPF patient biopsies may be crucial in supporting the high proliferation and migration events during the alveolar epithelium transformation. The strong induction by TGF-β1 of AQP1 expression specially observed in AECs (A549) in transition to a myofibroblast-like phenotype cell, or in the fibroblasts derived from an IPF patient (LL29), might be among the initial actions triggered by this potent fibrotic agent.

The initial injury which causes lesions in epithelial cells that lead to pulmonary fibrosis are unknown. For the very first time, we have demonstrated increased expression of AQP1 in type II alveolar epithelial cells in IPF patient samples. The increased expression of AQP1, its possible relationship with hyperplasia, and the subsequent insult of ATII remains unclear. Although deeper analysis are required to elucidate the role of AQP1 in the origin of IPF, our findings suggest AQP1 plays an important role in disease progression of IPF patients and should be further explored.

## Conclusions

The alveolar epithelium of a normal human lung does not express AQP1. However, our studies indicate that IPF patient's hyperplasic type II pneumocytes show an increase in AQP1 expression on their surface. We investigated the role of TGF-β1, in the epithelial to mesenchymal transition of alveolar epithelial cells and lung fibroblast in IPF patient samples and also confirmed the induction of AQP1 expression. Therefore, the appearance of hyperplastic cells and regulation of AQP1 are implicated in the physiopathology of IPF.

## Author contributions

AG-C and EA-O: Made all molecular biology experiments with culture cells; RS-S and NS-L: Carried out the immunoassays over patient's biopsies; JL-C, CG, MM-M, and JR: Selected, treated and diagnosed the patients included in the analysis; LG: Revised and interpreted immune results in patient's biopsies; ME and AG-C: Conceived and designed the experiments and wrote the manuscript; ME and JR: Contributed reagents, materials, and analysis tools. All authors read and approved the final manuscript.

### Conflict of interest statement

The authors declare that the research was conducted in the absence of any commercial or financial relationships that could be construed as a potential conflict of interest.
